# Protective effect of propolis in protecting against radiation-induced oxidative stress in the liver as a distant organ

**DOI:** 10.1038/s41598-024-72344-9

**Published:** 2024-09-18

**Authors:** Oztekin Cikman, Aziz Bulut, Seyithan Taysi

**Affiliations:** 1 Department of General Surgery, Faculty of Medicine, Yuzuncu Yıl University, Van, Turkey; 2 Department of General Surgery, Faculty of Medicine, Gaziantep University, Gaziantep, Turkey; 3 Department of Medical Biochemistry, Faculty of Medicine, Gaziantep University Medical School, Gaziantep, Turkey

**Keywords:** Cancer, Experimental study, Irradiation, Phenolic compounds, Propolis, Radiotherapy, Biochemistry, Cancer

## Abstract

Stresses caused by ionizing radiation can also damage tissues and organs through the circulatory system. In this study, we aimed to determine the radioprotective effect of propolis, a natural and powerful antioxidant product, against oxidative liver damage caused by cranial irradiation. Thirty-two male albino Sprague–Dawley rats, divided into four groups, were designed as sham group, irradiation (IR) group, propolis plus IR, control group of propolis. Biochemical parameters were measured in liver tissue of rats. While Total enzymatic superoxide scavenging activity (TSSA) and non-enzymatic superoxide scavenging activity (NSSA), glutathione peroxidase (GSH-Px) activities of all groups were statistically significantly higher than rats receiving only-irradiation, Glutathione-S-transferase (GST) activity in the IR group was significantly lower than in the sham control group and IR + propolis group. Superoxide dismutase (SOD) activity in the IR group was found to be significantly higher than both the sham control group and the propolis control group, but lower than the IR + propolis group. Malondialdehyde level and xanthine oxidase activity were higher in the IR group than in the other groups. Compared to the sham control group, in the group treated with propolis, a significant elevation in antioxidant parameters, specifically TSSA, NSSA, SOD, and GST activities, was noted, with corresponding increases of 32.3%, 23.2%, 47.6%, and 22.6%, respectively. Our findings show that propolis can be a radioprotective agent against ionized radiation damage by increasing antioxidant activity and reducing oxidant stress in liver tissue.

## Introduction

Radiotherapy (RT) stands as an indispensable approach in the management of cancer, a paramount and challenging ailment in contemporary times. The impacts of RT, encompassing both local and systemic ramifications, largely stem from the generation of free radicals induced by ionizing radiation. The excessive production of reactive oxygen species (ROS) and reactive nitrogen species (RNS) is identified as oxidative and nitrosative stress, respectively, inflicting numerous deleterious effects on living organisms. These species interact with cellular macromolecules, including DNA, cytoplasmic membranes, and functional proteins, thereby eliciting inflammation, cellular dysfunction, and ultimately leading to cell death^1,2^.

The cytotoxic and genotoxic effects resulting from oxidative and nitrosative damage induced by ionizing radiation have been demonstrated through studies, indicating that, in addition to the targeted cancer tissue, surrounding and distant normal tissues are also impacted by radiation^3^. Given that the liver, as an integral component of the systemic circulation, is susceptible to the side effects of radiation, it is imperative, akin to other organs, to shield the liver from the systemic repercussions of radiotherapy administered to any part of the body^3,4^. When the equilibrium between stresses and antioxidant defense mechanisms is disrupted, living organisms endeavor to counteract the oxidant effects and restore redox balance by activating or suppressing genes encoding protective enzymes, transcription factors, and structural proteins^3,5^.

Propolis, a substance produced by honeybees with an extremely complex chemical structure and antioxidant, anti-inflammatory and antitumoral properties, contains various chemical components such as polyphenols (flavonoid aglycones, phenolic acids and their esters, phenolic aldehydes, alcohols and ketones), sesquiterpene quinones, coumarins, steroids, amino acids, organic compounds and vitamins^3,6^. The components of propolis are responsible for many of its biological and pharmacological activities and have been reported to exhibit detoxification activity against the harmful effects of free radicals^3,7^. However, propolis may vary in composition and activity depending on its botanical sources and geographical origin^3^. In the analysis performed with a high-performance liquid chromatography device coupled to a diode array detector (HPLC–DAD), the ethanolic extract of Turkish propolis was reported to contain chrysin (641.33 µg/mL), caffeic acid phenethyl ester (630.67 µg/mL), pinocembrin (572.67 µg/mL), galangin (534.11 µg /mL), naringenin (372.39 µg/mL), as well as kaempferol, trans-cinnamic acid, caffeic acid, myricetin and quercetin^6^.

The results of our previous research have reported a decrease in antioxidant defense and an increase in oxidants in the lens, tongue, and brain tissues of rats subjected to total head irradiation. Conversely, in rats administered with propolis, we observed a reversal of this trend^1,7,8^.

Although the antioxidant properties of propolis have been investigated in various studies^9,10^, to the best of our knowledge, we have not come across a study specifically examining the protective effect of propolis against radiation-induced oxidative stress in the liver as a distant organ from the irradiated tissue. Therefore, our aim was to investigate whether propolis has protective effects against radiation-induced oxidative damage in the liver tissue of rats following systemic administration.

## Methods

### Animals and experiments

The study was conducted by ARRIVE guidelines and was carried out in the Departments of Medical Biochemistry and Radiation Oncology after receiving ethical approval from Gaziantep University Local Animal Ethics Committee (number 2017/2). Thirty-two male albino Sprague–Dawley rats, weighing 220 ± 25 g and aged 12–16 weeks, were utilized in the experiment. The rats were individually housed in LF-4 type cages (590X380X200 mm; length, width, and height, respectively) for a minimum of one week prior to radiation exposure. Standard laboratory chow and water were provided as the diet for the rats. Each cage housed eight rats in a windowless laboratory room with automated temperature control (22 ± 1 °C) and lighting controls (12 h light/12 h dark).

The rats were randomly divided into four groups, 8 rats in each group. Groups were organized as follows:

Irradiation (IR) Group: Rats in this group initially received 1 ml of saline via an orogastric tube. Subsequently, a single dose of 5 Gray (Gy) gamma irradiation was administered to the entire cranium. Rats in this group were orally administered 1 ml of saline daily for 10 consecutive days.

IR plus Propolis Group: Rats in this group were given propolis (80 mg kg-1 day-1) through an orogastric tube one hour before irradiation. This procedure was repeated daily for 10 days. Propolis was dissolved in dimethyl sulfoxide (DMSO) immediately before administration to the rats.

Propolis Control Group: Rats in this group did not receive propolis or irradiation. Instead, an equivalent volume of DMSO, in which propolis was dissolved, was orally administered via an orogastric tube. This process was carried out one hour before irradiation and repeated daily for 10 days.

Sham Control Group: Rats in this group did not receive propolis or irradiation.

### Ionizing radiation application

The research was conducted in accordance with ethical guidelines set by the local Ethical Committee. Prior to total cranial irradiation, all rats were anesthetized with 80 mg/kg ketamine hydrochloride (Pfizer Ilac, Istanbul, Turkey) and positioned in the prone position on a tray. Rats in the IR and IR plus Propolis groups were irradiated from anterior areas measuring 80 cm × 5 × 5 cm at a source-to-surface distance, utilizing a Cobalt-60 teletherapy unit (Theratron Equinox, MDS Nordion, Kanata, Ontario, Canada). Total cranium gamma irradiation was administered as a single dose of 5 Gy. Rats in the normal control and propolis control groups underwent placebo irradiation. The dose rate was 0.49 Gy/min, and the central axis dose was calculated at a depth of 0.5 cm.

### Biochemical analysis

#### Preparation of tissues for analysis

All rats were sacrificed by decapitation after anaesthesia with 50 mg of ketamine hydrochloride on the 11^th^ day under aseptic conditions and their livers were extracted. A volume of liver tissue, along with 9 volumes of physiological saline solution, was homogenized using an IKA-NERKE homogenizer device (Staufen, Germany).

The homogenate was centrifuged at 10,000 g for 1 h to remove residues. The clear supernatant from the top was collected, and all analyses were conducted on this fraction. All procedures were performed at 4 °C.

### Determination of TSSA, NSSA and SOD activities

Total enzymatic superoxide scavenging activity (TSSA) and non-enzymatic superoxide scavenging activity (NSSA) were evaluated in samples before and after the addition of trichloroacetic acid (TCA, 20%) as reported by Durak et al.^10^. Superoxide dismutase (SOD) activity was calculated from the difference between TSSA and NSSA^10^. Results were expressed as U/mg protein.

### Determination of GSH-Px, GST, XO and NOS activities

The activity of glutathione peroxidase (GSH-Px) was assessed following the method outlined by Paglia and Valentina^11^. Glutathione-S-transferase (GST) activity was determined as described by Habig et al.^12^. Xanthine oxidase (XO) activity was analyzed spectrophotometrically^13^. Nitric oxide synthase (NOS) activity was measured according to the described method ^14^. All results are expressed as U/mg protein.

### Determination of MDA levels

Malondialdehyde (MDA) was determined spectrophotometrically^15^, and the total thiobarbituric acid reactive substances (TBARS) were expressed as MDA. The results were expressed as nmol/g wet weight. NO^·^ levels were measured using the Griess reagent^16,17^, and the results were expressed as µmol/g wet weight. Protein content was determined as described^18^, and all measurements were conducted using a spectrophotometer (Shimadu U 1601, Japan).

### Statistical analysis

The data were subjected to statistical analysis using the Statistical Package for the Social Sciences for Windows (SPSS, version 23.0, Chicago, IL). The Kolmogorov–Smirnov test was employed to assess the normal distribution of continuous variables. Data were presented as mean ± SE (standard error). Differences between groups in normally distributed variables were evaluated using the ANOVA test, while the Kruskal–Wallis H test was utilized for data that did not exhibit a normal distribution. Statistical significance was considered when the p-value was less than 0.05.

## Results

### Antioxidant parameters

Antioxidant parameters measured are given in Table 1. TSSA, NSSA, GSH-Px activities of all groups were found to be statistically significantly higher when compared to rats that received only irradiation. GST activity in the IR group was significantly lower than the sham control group and IR + propolis group. SOD activity in the IR group was found to be significantly higher than both the sham control group and the propolis control group, but lower than the IR + Propolis group. In the group given propolis, a significant increase in antioxidant parameters TSSA, NSSA, SOD and GST activities was found to be 32.3%, 23.2%, 47.6% and 22.6%, respectively. This observed increase confirms our hypothesis that propolis increases antioxidant defense and prevents oxidative stress in rats receiving ionizing radiation.

**Table 1:** Antioxidant parameters measured in the liver tissue of the rats.

	Sham control group	Control group of propolis	IR group	IR plus propolis group
TSSA (U/mg protein)	2383.6 ± 80.7^a,¥^	2360.9 ± 43.7^a,¥^	2062.2 ± 96.5	3152.5 ± 134.9
NSSA (U/mg protein)	1745.2 ± 111.2^ k, &^	1650.8 ± 46.0^d,≠^	1124.0 ± 91.9	2150.4 ± 113.5^ k^
SOD (U/mg protein)	763.4 ± 42.0^c,¥^	735.2 ± 30.9^b,¥^	938.2 ± 37.0	1127.2 ± 59.2^b^
GSH-Px (U/mg protein)	12.0 ± 0.3^a^	12.5 ± 0.6^b^	10.6 ± 0.2	12.7 ± 0.5^b^
GST (U/mg protein)	175.8 ± 8.0^a^,*	146.7 ± 4.8^¥^	144.0 ± 5.9	215.6 ± 17^ k^

a: *p* < 0.05, b: *p* < 0.005 c: *p* < 0.01, d: *p* < 0.001, k: *p* < 0.0001 vs. IR group.

*: *p* < 0.05, &: *p* < 0.005 ≠ : *p* < 0.001, ¥: *p* < 0.0001 vs. IR plus Propolis group.

IR: Irradiation.

TSSA: Total enzymatic superoxide scavenging activity.

NSSA: Non-enzymatic superoxide scavenging activity.

GSH-Px: Glutathione peroxidase.

GST: Glutathione-S-transferase.

### Oxidative parameters

**Fig. 1:**
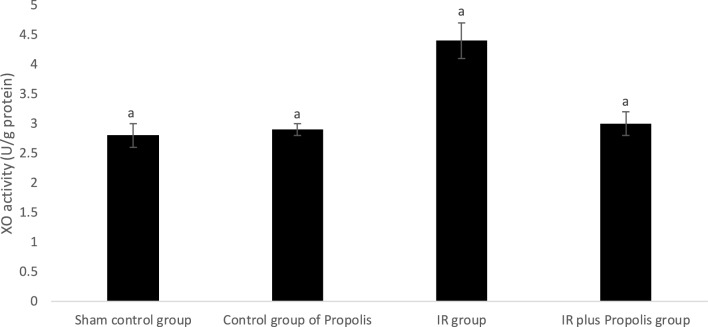
Xanthine oxidase activities measured in the liver of the rats.

**Fig. 2:**
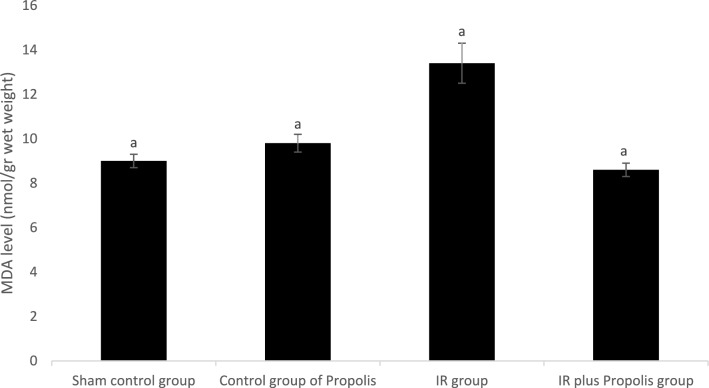
Malondialdehyde levels measured in the liver tissue of the rats.

MDA level and XO activity in liver tissue were found to be statistically significantly higher in the IR group compared to the other groups (Table 2). Significant increases in MDA levels (Fig. 1), a marker of lipid peroxidation, and in the activity of XO (Fig. 2), an oxidant enzyme, are also important indicators of oxidative stress caused by ionizing radiation. This confirms our hypothesis that components contained in propolis reverse oxidative stress in rats receiving ionizing radiation.

**Table 2:** Oxidant parameters measured in the liver tissue of the rats.

	Sham control group	Control group of propolis	IR group	IR plus propolis group
MDA (nmol/gr wet weight)	9.0 ± 0.3^a^	9.8 ± 0.4^a^	13.4 ± 0.9	8.6 ± 0.3^a^
XO (U/g protein)	2.8 ± 0.2^a^	2.9 ± 0.1^a^	4.4 ± 0.3	3.0 ± 0.2^a^

a: p < 0.0001 vs. IR group.

MDA: Malondialdeyde.

XO: Xanthine oxidase.

## Discussion

Exposure to ionizing radiation, which causes a significant increase in physiological and metabolic processes and oxidation chain reactions, also leads to significant increases in ROS, RNS production and ultimately lipid peroxidation, which cause stresses^3^. Propolis, which contains many important compounds, including aliphatic and phenolic acids, phenolic esters and flavonoids, has been carefully studied for its potential chemical inhibitory properties and its pharmacological safety has been the subject of studies^1,19^. For example, agents such as apigenin, ellagic acid, caffeic acid, caffeic acid phenethyl ester, kaempferol, naringenin, galangin, pinocembrin, quercetin can be given^3^. Scientific investigations have indicated that these plant polyphenols impede pathways associated with treatment resistance and can be utilized to enhance the sensitivity of tumor cells to chemotherapeutic agents and radiation therapy^3,20–22^.

In our findings, a statistically significant increase in oxidative stress markers was detected in liver tissues of rats exposed to gamma rays compared to other groups. In contrast, there was no such increase in rats receiving both propolis and ionizing radiation. This indicates that ionizing radiation has the potential to induce oxidative stress in remote tissues and organs. It supports our hypothesis that propolis protects tissues and organs away from the stresses caused by ionizing radiation.

### Effects of propolis on oxidative stress parameters in liver

Interestingly, MDA values, which are a marker of lipid peroxidation, were found to be numerically lower in the propolis-administered group than in the control groups. Interestingly, it was found that the MDA values in the group given propolis were numerically slightly lower than the control groups. XO, an oxidizing enzyme, produces the superoxide anion radical (O_2_^·–^), which is a potentially highly toxic radical for cellular structures. In stress situations, lipid peroxidation reactions begin with the oxidation of polyunsaturated fatty acids (PUFA) in membrane structures and are one of the oxidative conversions to products such as MDA, often measured as thiobarbituric acid reactive substances (TBARS) or lipid peroxides^23^. Our results were in agreement with previous studies that oxidative stress induced by ionizing radiation was reversed by propolis^23,24^.

### Effects of propolis on antioxidant parameters in liver

Superoxide dismutase neutralizes O_2_^·–^ radicals and protects living organisms against radical damage by converting O_2_^·–^ radicals to hydrogen peroxide (H_2_O_2_). GSH-Px neutralizes H_2_O_2_ by converting it to molecular oxygen and water. Under normal conditions, this cooperation between SOD and GSH-Px enzymes was impaired only in irradiated rats. Contrary to the significant increase in SOD activity in the IR group, the decrease in GSH-Px activity may be one of the main causes of oxidant stress in the IR group. Our findings show that propolis prevents oxidative stress caused by ionizing radiation and also leads to a statistically significant increase in antioxidant enzyme activities. This supports our hypothesis that propolis acts by a mechanism that increases antioxidant activity under stress conditions induced by ionizing radiation.

Propolis has been reported to contain more than 300 chemical compounds^25,26^. Of these components, CAPE, which has immunomodulatory, antitumoral, cytotoxic, antimetastatic, anti-inflammatory and antioxidant properties and suppresses lipid peroxidation by inhibiting lipoxygenase activities^4^, and Quercetin, which scavenges free radicals, chelates metal ions, XO, inhibits lipid peroxidation, shows important activities by reducing oxidative stress^3^.

As mentioned above, the other mechanism of propolis is to neutralize the electrons of radicals with the strong scavenging effect of the antioxidant components it contains.

### Strengths and limitations of the research

One of the strengths of the current study is that, since it is an animal experiment, it does not have confounding factors such as comorbidities that are often seen in humans. In addition, direct use of liver tissue in the evaluation of oxidative damage is another advantage. The lack of histological evaluation is one of the biggest limitations.

While biochemical analyses suggest that propolis provides radioprotective effects against oxidative damage in the liver tissue of irradiated rats, it would be judicious to complement these findings with histological evaluations. Furthermore, an ideal radioprotective agent would exhibit selectivity for normal tissues, sparing tumor tissues affected by radiotherapy. Regrettably, the current study lacks data for such a comparison with propolis, presenting an additional limitation. The study, structured as an animal experiment, inherently restricted parameters to a limited number of samples and a small group of rats. Despite a statistical evaluation, conducting analogous studies with larger sample sizes would be advantageous.

## Conclusions

In this study, we found that oxidative stress increased and antioxidant defense system decreased in liver tissue of irradiated rats compared to other groups. To our knowledge, this is the first study to report the radiation protective effect of propolis in rat liver tissue. This natural substance shows that it protects liver tissue against stress by reducing lipid peroxidation formation, increasing antioxidant activity and also preventing free radical formation. These findings suggest that it is likely to be a valuable agent for use as an antioxidant and/or protection against gamma radiation in cancer patients treated with radiotherapy. Nevertheless, additional research is essential to implement the utilization of natural compounds for clinical protection against ionizing radiation, particularly in the treatment of cancer patients undergoing radiotherapy.

## Data Availability

The datasets used and/or analysed during the current study available from the corresponding author on reasonable request.
